# Simvastatin regulates the proliferation, apoptosis, migration and invasion of human acute myeloid leukemia cells via miR-19a-3p/HIF-1α axis

**DOI:** 10.1080/21655979.2021.1999552

**Published:** 2021-12-11

**Authors:** Hua Tian, Tiao Qiang, Jinbo Wang, Li Ji, Bo Li

**Affiliations:** a Department of Blood Transfusion, Baoji People’s Hospital, Baoji City, Shanxi Province, China; b Department of Laboratory, Yanan University Hospital, Yanan City, Shanxi Province, China; c Department of Laboratory, Baoji People’s Hospital, Baoji City, Shanxi Province, China; d Department of Blood Transfusion, Hanzhong People’s Hospital, Hanzhong City, Shanxi Province, China

**Keywords:** Simvastatin, AML, cell proliferation, miR-19a-3p, HIF-1α

## Abstract

Statins are mainly used to lower plasma cholesterol level. In addition, the anti-leukemia effect of statins has been reported, but the mechanism remains unclear.

This study aimed to explore the bioregulation of simvastatin and its mechanism in acute leukemia cell lines. Cell viability was detected by CCK-8 analysis. Apoptosis was detected through flow cytometry. Cell invasion and migration both were observed by transwell and wound healing separately. RT-qPCR and Western blot were used for determination of genes and proteins. We found that that simvastatin could regulate the biological functions of acute myeloid leukemia (AML) cells, including its proliferation, migration, invasion and apoptosis, which may be carried out by down-regulating miR-19a-3p. Overexpression of miR-19a-3p had the opposite effect in AML cells, suggesting simvastatin-inhibited AML by reducing miR-19a-3p expression. Following researches showed that HIF-1α was directly regulated by the target of miR-19a-3p. Simvastatin could reverse the adverse effects caused by miR-19a-3p mimics. Conversely, the increased expression of Mcl-1, the inhibition of caspase-3 could promote the growth of AML cells. In conclusion, simvastatin could inhibit the proliferation, migration, invasion and promote apoptosis in AML cells through miR-19a-3p/HIF-1α axis.

## 1. Introduction

Acute myeloid leukemia includes a series of heterogeneous hematologic malignancies with features of clonal myeloid cell expansion in marrow, blood and other tissues. According to the American Cancer Society, there are about 21,380 new cases and 10,590 deaths of AML [1]. Although modern chemotherapy has made progress in the treatment of AML, the prognosis of patients is still poor [2,3]. Therefore, further understanding of the relevant mechanisms of AML is beneficial to the emergence of innovative treatment methods and the improvement of the prognosis of patients.

The statins are widely used to lower plasma cholesterol levels. In addition, in recent years, lots of studies have shown that statins have additional effects in different types of solid cancer cells, including anti-proliferation, pro-apoptosis and anti-metastasis [4–8]. It has also been reported that statins can induce a variety of human AML cell lines and human AML cell death in vitro [9–12]. However, the exact mechanism of statins induced cytotoxicity remains unclear. Recent studies have shown that not all human AML samples show the same cytotoxicity to statins [13]. As early as 15 years ago, studies have shown that Simvastatin can selectively inhibit the growth of primary acute myeloid leukemia cells [14], and was used as a clinical trial drug for leukemia patients [15]. In addition, other studies have shown that simvastatin, an inhibitor of 3-hydroxy-3-methylglutaric acid CoA reductase (HMG-CAR) synthase, induces AML cell death through a similar apoptosis process and has synergistic effect with F10 [16]. Moreover, simvastatin combined with tepifanib shows additive cytotoxicity on AML cell lines [17]. Scientists concluded that simvastatin had a major anti-proliferative effect on AML blasts in vitro [12]. Furthermore, the combination of simvastatin and cytarabine also has a collective anti-proliferation effect [18]. On the other hand, animal experiments also indicated that simvastatin was critical to inhibit proliferation of AML cells in severe combined immunodeficiency (SCID) mice [19]. These findings provide new ideas for basic and clinical research of leukemia. However, the underlying mechanism of simvastatin in AML has not been completely clarified.

MicroRNAs (miRNAs) are a single chain nonencoding RNA, consisted of 18–23 nucleotides, which directly combined to 3ʹ-UTRs of the target gene and regulate gene expression after transcription [20–22]. Many researches have proved miRNAs can adjust the occurrence and development of tumor [23–25]. It is reported that many miRNAs are abnormally expressed in AML [26–28], which suggests that miRNAs may be potential therapeutic targets for AML. MiR-19a-3p is a tumor suppressor target, which can inhibit the growth and induce apoptosis of a variety of tumor cells [29–32]. Yet, the critical meaning of miR-19a-3p in AML mentioned in this study has not been defined.

This study aims to explore the bioregulation of simvastatin in acute leukemia cell lines, the expression of miR-19a-3p and HIF-1α level in AML, the molecular mechanism of miR-19a-3p/HIF-1α in simvastatin inhibiting AML, to lay a theoretical foundation for possible clinical application in the future.

## 2. Materials and Methods

### 2.1. Cell culture and reagents

KG-1 (human AML cells) and HS-5 (normal bone marrow cell lines) were obtained from Shanghai binsui Biotechnology Co., Ltd. KG-1 and HS-5. The cell was cultured in RPMI1640 medium (GIBCO) containing 10% FBS (Gibco) at constant temperature (37 °C) and 5% CO_2_. The cells were seeded 100 μL (5 × 10^3^ cells) in 96-well plates. The cells were incubated for 24 hours and then treated with 0.4 mm simvastatin for 3 days. Subsequently, we treated KG-1 cells with simvastatin, and then transfected with miR-19a-3p mimics or not to explore the significance of miR-19a-3p/HIF-1α axis on the growth of AML cells after the treatment of simvastatin. Antibodies against HIF-1α, cleaved caspase-3, Mcl-1, and goat anti rabbit antibodies were purchased from CST (CST, USA). Simvastatin (Merck) was dissolved in dimethyl sulfoxide (10 mM) to reserve.

### 2.2. Oligonucleotide design and synthesis

MiR-19a-3p mimic oligonucleotide was designed and then chemically synthesized by Shanghai Gemma company. The cells were seeded into the basic cell culture medium without antibiotics and cultured in a constant temperature incubator at 37 °C and 5% CO_2_. Transient transfection was used to promote the expression of miR-19a-3p. The operation steps of transfection were completely carried out according to the instructions. They were divided into mimic group (cells transfected with miR-19a-3p mimics), negative control group (miR-NC) and untreated control group.

### 2.3. Reverse-transcription quantitative polymerase chain reaction

The total RNA of cells in each group was extracted with Trizol reagent (Merck, USA), and then 2 μg RNA was reverse transcribed into cDNA by kit (Takara company of Japan). MiR-19a-3p levels, HIF-1α levels and GAPDH levels were detected through SYBR Green quantitative PCR [33]. All PCR tests were performed with S1000 Thermal cycler PCR system (BioTek, USA). GAPDH was used as an internal parameter. 2^−ΔΔCt^ method served to analyze the expression of related genes.

### 2.4. CCK8 assay

Cell suspension (100 μL/well) was prepared first [34]. After cultured for 6 h, 10 μL CCK8 (Biyuntian Biotechnology Co., Ltd.) were added to each well after incubation in a 5% CO_2_ incubator for 2 hours, 5% CO_2_ incubator was used to determine absorbance (a value) by enzyme linked immunosorbent (ELISA) at 450 nm. Five multiple holes were set in each group to take the average value.

### 2.5. Flow cytometry analysis of apoptosis

KG-1 and HS-5 cells were inoculated in 6-well plate at 1 × 10^6^ cells/mL (2 ml in total). They were added with 10 μmol/L and 20 μmol/L V-9302, respectively. After 48 hours of culture at 37 °C and 5% CO_2_, 400 μL binding buffer was used to resuspend cells, add 5 μL annexin V-FITC solution (shanghai Beibo Biotechnology Co., Ltd,) gently mix well, incubate in dark at 2–8 °C for 15 min, and then add 10 μL PI dye, mix it gently again. After incubation at 2–8 °C for 5 min, the cells were detected by upflow cytometry within 1 h and analyzed by Flowjo-V10 [35].

### 2.6. Cell migration and invasion assay

The transfected cells were added into 100 μL serum-free medium (1 × 10^4^ cells/well). The inferior chamber was full of medium containing 10% fetal bovine serum (FBS). After 24 h intervention, the cells were counted by microscope (Olympus) to determine the specific situation of cell invasion or migration [36].

### 2.7. Luciferase reporter assay

Luciferase reporter plasmids (pmiR-HIF-1α-3′-UTR wt, pmiR-HIF-1α-3ʹ-UTR mut) were obtained from GenePharma. Cells were seeded in 24-well plates, the density was 4 × 10^5^ cells/well. According to the manufacturer’s protocol, miR-Nc or miR-19a-3p mimics were mixed with pmiR- HIF-1α-3ʹ-UTR via Lipofectamine 2000α-3ʹ-UTR WT or pmiR-IGF-1 R-3ʹ-UTR MUT were co-transfected into cells. Luciferase activity was detected by double Luciferase Report Analysis System (Promega company, USA). The results suggest that miR-19a-3p and 3′- UTR of HIF-1α is in a direct interaction [34].

### 2.8 Protein extraction and Western blot

Protein was extracted by standard process [37], electrophoresed and transferred to PVDF membrane. Nonspecific bands were removed with TBST buffer (5% skimmed milk) at room temperature for 1 h. PVDF membrane was incubated in the first antibodies at 4 °C overnight, the dilution of primary antibody was 1:1000. GAPDH was regarded as a reference. The membranes were blocked with TBST at about 25°C for 15 minutes. The HRP labeled secondary antibodies were diluted with TBST at a ratio of 1:10,000 and NC membrane was incubated in shaker for 1 h. The membranes were blocked with TBST for 15 minutes. At the end of the experiment, liquid A and liquid B in ECL luminescent agent were mixed in a ratio of 1:1, then the ECL luminescent solution was transferred to NC film with protein surface, reacted for 2 min at room temperature, dried ECL luminescent solution, exposed, developed, fixed and scanned in dark room.

### 2.9. Statistical analysis

All data of this study are represented by mean ± standard deviation, and parallel experiments were conducted for three times or more. *T*-test was used to compare the two groups of data; one-way ANOVA was used for inter group comparison. GraphPad Prism was used as analysis software. P < 0.05 was considered the difference to be significant.

## 3. Results

Acute myeloid leukemia is one of the most popular malignancy globally. Understanding the pathogenesis and progression mechanism of AML may offer us new strategies for integrated treatment of AML patients. Simvastatin has been reported inducing a variety of human AML cell lines and human AML cell death in vitro. However, its mechanisms need further elucidation. In the present study, we conducted a series of in vitro assays, aimed to explore the molecular mechanism of miR-19a-3p/HIF-1α in simvastatin inhibiting AML, to lay a theoretical foundation for possible clinical application in the future.

### 3.1. High miR-19a-3p and HIF-1α expression in KG-1 cells

**Figure 1.**
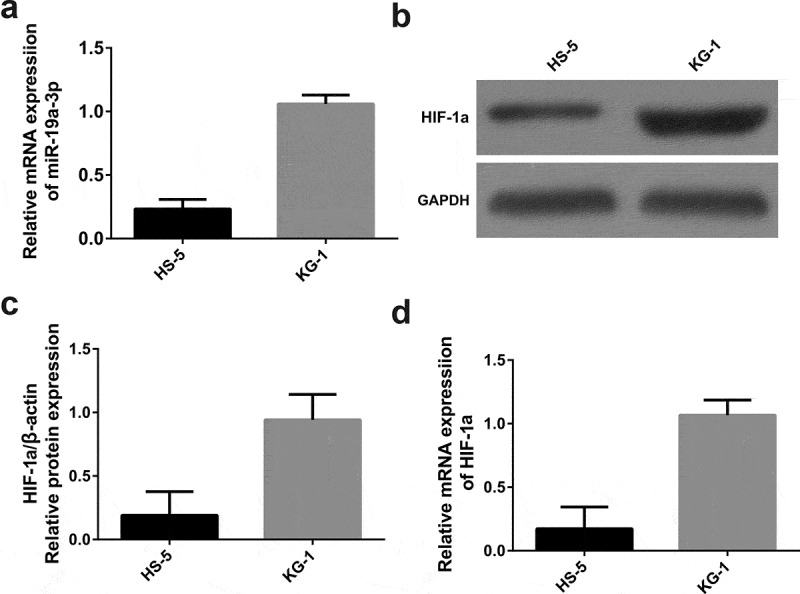
miR-19a-3p and HIF-1α expression. (a) The levels of HIF-1α were determined by WB (b) and qRT-PCR (c) MiR-19a-3p mRNA level was detected through qRT-PCR. (d) Results pointed out miR-19a-3p and HIF-1α levels in KG-1 cells were higher than HS-5 cell lines

Comparing HS-5 cells with KG-1 cells, miR-19a-3p expression was determined in two groups through RT-qPCR. As is shown in Figure 1(a), compared with HS-5, the expression of miR-19a-3p in KG-1 group was obviously increased (*P* < 0.05), indicating that overload of miR-19a-3p would lead to the appearance of AML.

### 3.2. Simvastatin inhibits miR-19a-3p and HIF-1 α expression

**Figure 2.**
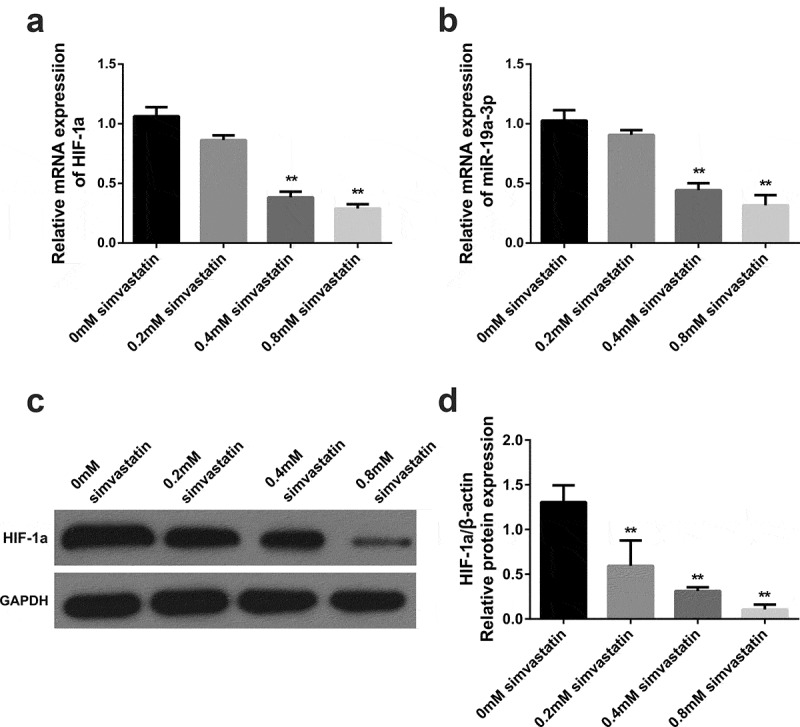
Effects of different concentrations of simvastatin on miR-19a-3p/HIF-1α in KG-1. Different concentrations of simvastatin were added to intervene KG-1 for 24 hours. (a–b) HIF-1α and miR-19a-3p mRNA levels were determined through qRT-PCR. (c–d) HIF-1α protein level was identified by WB

MiR-19a-3p and HIF-α levels were decreased in KG-1 when it was intervened with different concentrations of simvastatin. These differences were dose-dependent. As shown in Figures 2, 0.4 mM simvastatin significantly reduced miR-19a-3p and HIF-1α expression in KG-1 cells. Thus, 0.4 mM simvastatin was selected for the subsequent studies to explore the effect of simvastatin on the biological behavior of acute myeloid leukemia.

### 3.3. Simvastatin regulates the proliferation, apoptosis, migration and invasion of AML cells by inhibiting the expression of miR-19a-3p

**Figure 3.**
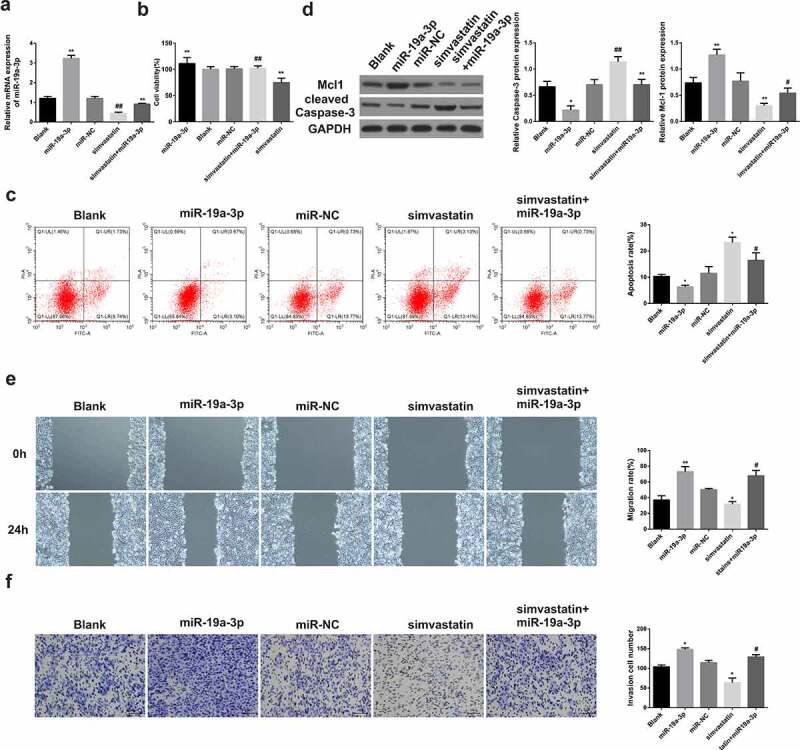
Simvastatin induces miR-19a-3p expression in AML cells to regulate the proliferation, apoptosis, migration and invasion. (a) qRT-PCR served to identify miR‐19a‐3p mRNA of AML cell lines. (b) Cell viability was analyzed by CCK-8. (c) Apoptosis analysis was identified through Flow Cytometry. (d) Apoptosis protein levels were performed by WB. (e) The migration and invasion were observed by transwell and wound healing. Compared with control group, *P < 0.05, **P < 0.01. Compared with the simvastatin group, ^#^*P* < 0.05, ^##^*P* < 0.01

After transfection with miR-19a-3p, miR-19a-3p levels were increased in KG-1 cells, but were decreased after treatment with simvastatin (Figure 3(a)). After miR-19a-3p transfection, the proliferation (Figure 3(b)), migration (Figure 3(e)) and invasion (Figure 3(f)) of KG-1 cells were enhanced, while the apoptosis (Figure 3(c)) was significantly inhibited, the expression of Mcl-1 increased and caspase-3 were decreased (Figure 3(d)). However, the situation in KG-1 cells treated with simvastatin was opposite to that in KG-1 transfected with miR-19a-3p. In the cells treated with simvastatin and transfected with miR-19a-3p, the effects of simvastatin on the proliferation, apoptosis, migration and invasion of KG-1 were eliminated by miR-19a-3p transfection. These results made clear that simvastatin can regulate the biological function of AML cells by decreasing miR-19a-3p expression.

### 3.4. MiR-19a-3p directly targets HIF-1α

**Figure 4.**
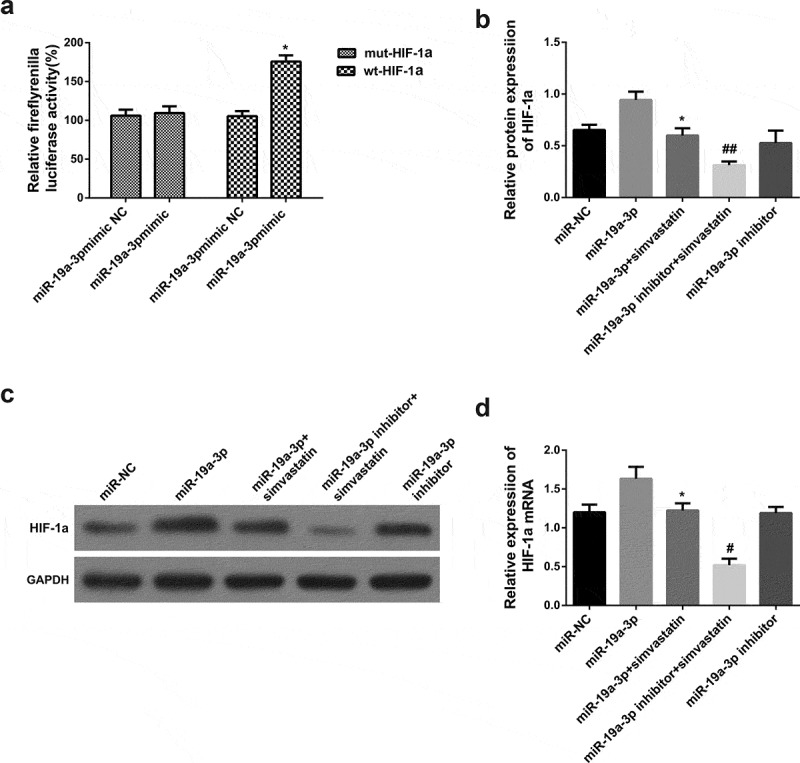
miR-19a-3p directly targets HIF-1α. (a) Luciferase activity was detected by luciferase gene reporting experiment. (b) qPCR severed to identify HIF-1α mRNA levels. (c–d) HIF-1α protein level was performed through WB. Statistical analysis: compared with miR-19a-3p group, *P < 0.05, **P < 0.01. Compared with the miR-19a-3p inhibitor group, ^#^*P* < 0.05, ^##^*P* < 0.01

MiRNA regulated downstream gene expression by combining 3ʹ-UTR. There is a relationship between occurrence and development of AML and HIF-1α, so further experiments on HIF-1α were carried out. Luciferase activity experiment was used to prove the hypothesis. According to the final results, miR-19a-3p can increase HIF-1α in KG-1 cells, the difference was statistically significant. However, there were no notable changes in the 3ʹ-UTR mut luciferase plasmid (Figure 4(a)), indicating miR-19a-3p was relevant to HIF-1α. There is a direct interaction between 3ʹ-UTR. HIF-1α levels was further determined in several groups (miR-NC group, miR-19a-3p group, miR-19a-3p and simvastatin group, miR-19a-3p inhibitor group, miR-19a-3p inhibitor group and simvastatin group). Results indicated that the levels of HIF-1α in miR-19a-3p group was significantly increased than that of miR19a-3p and simvastatin group. Compared with miR-19a-3p group, HIF-1α mRNA expression of inhibitor group and simvastatin group decreased significantly (Figure 4(b-d)). They made clear that miR-19a-3p is in direct contact with HIF-1α, and simvastatin regulate growth of tumor cells through this mechanism possibility.

### 3.5. Simvastatin regulates the proliferation, apoptosis, migration and invasion of human acute myeloid leukemia cells via blocking HIF-1α by miR-19a-3p

**Figure 5.**
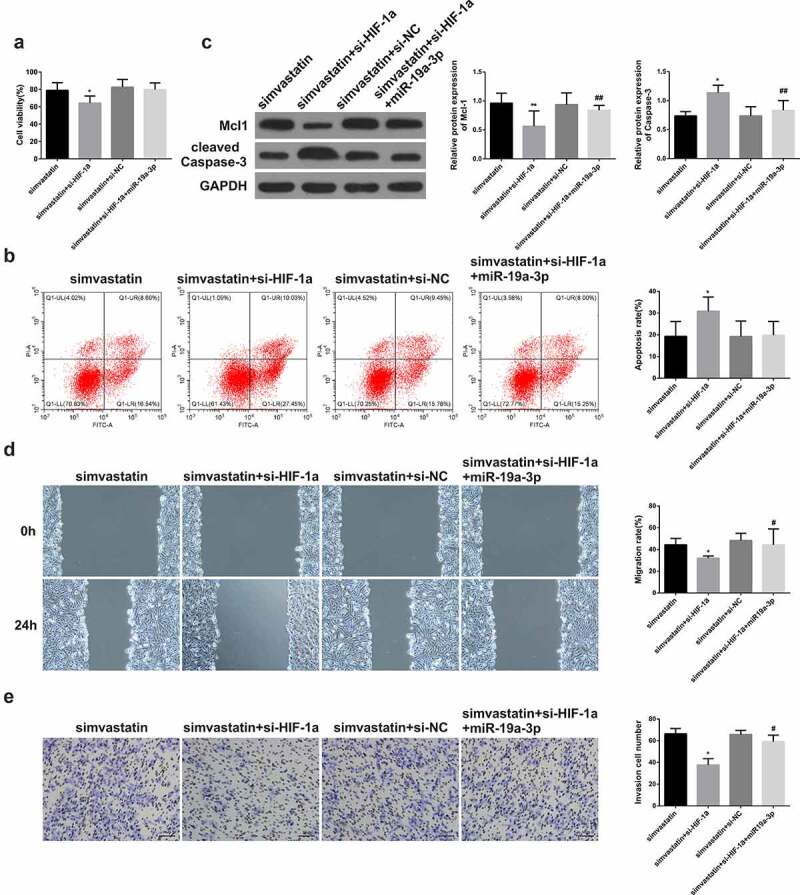
Simvastatin regulates the proliferation, apoptosis, migration and invasion of human acute myeloid leukemia cells via blocking HIF-1α by miR-19a-3p. (a) Cell proliferation was detected through CCK-8. (b) Cell apoptosis analysis was observed via Flow Cytometry. (c) The levels of apoptosis protein of Mcl-1, caspase-3 were performed by WB. (d-e) The migration and invasion were detected by wound healing assay and transwell separately. Statistical analysis: compared with simvastatin group, *P < 0.05, **P < 0.01. Compared with the simvastatin+si-HIF-1α group, ^#^*P* < 0.05, ^##^*P* < 0.01

For the sake of proving the role of miR-19a-3p/HIF-1α axis on the simvastatin-mediated regulation on biological function of AML cells, KG-1 cells were treated with simvastatin and silenced the expression of HIF-1α, then transfected with miR-19a-3p or not. CCK-8 result showed that simvastatin could inhibit cell proliferation, while this effect was enhanced by silencing HIF-1α. Conversely, this effect was reversed when it was transfected with miR-19a-3p (Figure 5(a)). Besides, treatment with simvastatin and si-HIF-1α, then transfection of miR-19a-3p in KG-1 obviously reduced cell apoptosis, which could be induced by simvastatin and si-HIF-1α alone, and the difference was statistically significant (Figure 5(b)). WB results revealed simvastatin significantly decreased Mcl-1 levels in KG-1 cells, while caspase-3 levels increased. Moreover, si-HIF-1α strengthened the changes in Mcl-1 and cleaved caspase-3 protein expression levels. Conversely, this effect was reversed when it was transfected with miR-19a-3p (Figure 5(c)). Transwell invasion test results showed simvastatin could inhibit cell migration and invasion, while this effect was enhanced by silencing HIF-1α. Conversely, this effect was reversed when it was transfected with miR-19a-3p (Figure 5(d,e)).

## 4. Discussion

Acute myeloid leukemia is the most dangerous disease by causing millions of deaths worldwide. Despite rapid advancement in research of therapeutics including stem cell therapy as well as CAR-T cell therapy [38,39], the prognosis is still poor for most patients, with a five-year survival rate lower than 50% [40]. Comprehensive knowledge about AML would allow to design better therapeutic systems. Statins represented by simvastatin are a kind of cholesterol lowering drugs, which mainly play a role by inhibiting the level of HMG-CAR, which is related to the conversion of HMG-CA to mevalonate [41]. The anti-leukemia effect of simvastatin on human AML cells in vitro is especially obvious in the AML cell line with NRASG12D mutation [42]. Combining tipifarnib and simvastatin was successful in targeting RAS/ERK signaling and inducing apoptosis in leukemia cells [43]. In our study, the results of CCK-8 and flow cytometry showed simvastatin played a role by inhibiting the growth of tumor cells, promotion of apoptosis, and inhibition of cell migration and invasion in animal experiments. Besides, we also found expression of cleaved caspase-3 was up-regulated by simvastatin, while the expression of Mcl-1 decreased.

Previous studies showed many miRNAs are abnormally expressed in the occurrence and development of AML. Zhu found miR-9 decreased in AML patients with adverse prognosis [44]. In addition, miR-21 is up-regulated in subjects with acute myeloid leukemia and is associated with a low-risk state [45]. Therefore, reducing the expression of miR-21 has been proposed as a potential treatment for AML. Although a lot of studies have shown that simvastatin has anti-leukemia effect, the role of miR-19a-3p in the treatment of AML with simvastatin was still unclear [38,39]. miR-19a-3p is likely to be a therapeutic target in many cancers such as CRC and prostate cancer [46,47]. MiR-19a-3p could regulate the Forkhead box F2-mediated Wnt/β-catenin signaling pathway and affect the biological functions of colorectal cancer cells. In this study, we showed that miR-19a-3p was significantly overexpressed in AML cell lines. Simvastatin inhibited the growth of AML cancer cells, and this effect was inhibited by reducing the expression of miR-19a-3p. Therefore, miR-19a-3p may provide new ideas and directions for the therapy of AML.

HIF-1α is of great significance to cell proliferation, apoptosis and migration, and can be used as a downstream gene in this study [48]. HIF-1α have been reported to be overexpressed in patients with AML, and is associated with poor survival in normal karyotype adult acute myeloid leukemia [49]. Targeting HIF-1α can increase apoptosis of AML cells. Therefore, it is essential to identify effective HIF-1 inhibitors. In this study, the relationship of miR-19a-3p and HIF-1α were detected by luciferase reporter gene assay. Most importantly, we confirmed that simvastatin has the function of regulating cell biological function by miR-19a-3p/HIF-1α signaling pathway.

## 5. Conclusion

In conclusion, our findings indicated that simvastatin inhibit AML progression by down-regulating miR-19a-3p and HIF-1α expression. Our research pointed out the molecular mechanism of simvastatin in the treatment of AML, suggested miR-19a-3p/HIF-1α may be promoted as a promising molecular target for the diagnosis and therapy of AML.

## Highlights

- Simvastatin regulates the proliferation, apoptosis, migration and invasion of human acute myeloid leukemia cells by inhibiting the expression of miR-19a-3p.
- HIF-1α was directly regulated by the target of miR-19a-3p.
- Simvastatin showed anti-leukemia effects via blocking HIF-1α by miR-19a-3p.
